# Granulocytic Sarcoma Presenting as Atypical Mastoiditis with Facial Paralysis: Description of a Case

**DOI:** 10.1155/2011/191852

**Published:** 2011-09-07

**Authors:** M. Crovetto, J. A. Márquez, C. Ereño, J. Elexpuru, R. Crovetto, A. Martinez

**Affiliations:** ^1^Otolaryngology Department, Hospital de Basurto, University of the Basque Country, Bilbao, Spain; ^2^Haematology Department, Hospital de Basurto, Avenida de Montevideo s/n, 48013 Bilbao, Spain; ^3^Patology Department, Hospital de Basurto, Bilbao, Spain; ^4^Neurosurgery Department, Hospital de Basurto, Bilbao, Spain; ^5^Dentistry Department, Clinica Bilbao, Simon Bolivar 7, 48010 Bilbao, Spain; ^6^Nursing Department, University of the Basque Country, Barrio Sarriena s/n, 48940 Leioa, Spain

## Abstract

We describe a case of temporal granulocytic sarcoma in a 26-year-old patient after apparent molecular remission of an acute myeloid leukaemia. He complained of otodynia with hearing loss and facial paralysis on the right side. He was treated with chemotherapy and self-transplant haematopoietic stem cells. He was cured clinically, molecular remission of the haematological processes was achieved, and he remained asymptomatic for three years. Facial paralysis and hearing loss associated with temporal GS should be treated with chemotherapy. Aggressive surgery may complicate the clinical course of the disease and it should be avoided.

## 1. Introduction

Granulocytic sarcoma (GS) or myeloblastoma is a malignant tumour originated form myeloid cells located outside the bone marrow. It can appear anywhere, including bone, periostium, skin, and lymph nodes [1, 2].

Granulocytic sarcoma is generally associated to acute myeloblastic leukaemia, either as its first clinical manifestation, tissue expression of this disease, or initial symptom of its recurrence [1, 2]. It can also appear as the first symptom or in association with other haematological neoplasiae, such as chronic myeloid leukaemia, polycytaemia vera rubra, hypereosinophilic syndrome [1, 2].

We report the case of a young patient suffering from granulocytic sarcoma (GS) of his temporal bone that presented to us with facial paralysis and other symptoms suggestive of atypical mastoiditis.

## 2. Case Report

26-year-old male patient presented to us complaining of progressive and rapid hearing loss, severe pain in the right ear and facial asymmetry. He had no medical history of note other than M-2 subtype acute myeloblastic leukaemia diagnosed seven months earlier (April 2003). Cytogenic studies of his bone marrow had shown the presence of *t* (8; 21) (q22; q22) in 100% of the cells in metaphase. Molecular RNA studies of the same sample had shown the existence of the AML1-ETO chimerical transcript. The patient had undergone chemotherapy with Idarubicin, Cytarabine, and Etoposide. Complete cytological and immunological remission was achieved after the first cycle. The patient lacked of a histocompatible family donor, and, therefore, the treatment was continued with two more cycles of Mitoxantrone and Cytarabine in high doses (HDAC). Following treatment, the patient achieved clinical and molecular remission.

**Figure 1:**
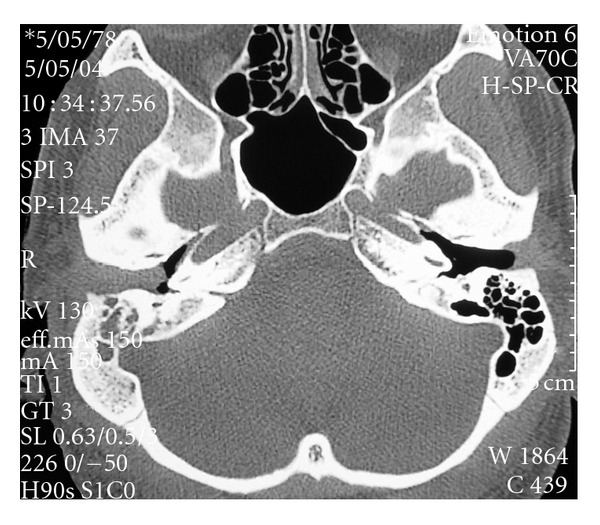
Axial CT of both temporal bones. The mastoid air cells on the right appear filled with a soft tissue density as well as the external auditory canal. The middle ear is filled with air.

On examination, the patient temperature was normal and showed tumefaction and retroauricular pain, as well as, painful bulging in the upper and posterior walls of the external auditory canal. The eardrum could hardly be seen but it looked opaque and swollen. The patient also had a grade III House-Brackmann facial paralysis on the right side. Eardrum paracentesis was performed but only blood stained fluid was retrieved, and this procedure did not improved the patient's clinical condition. Audiometry showed transmission hearing loss on the right ear. Full Blood Count and erythrocyte sedimentation rate were normal. Computed tomography (CT) with and without I.V. contrast demonstrated occupation of the right mastoid cells and the external auditory canal by a soft tissue density mass. Attic was also filled with soft tissue density mass but no bone or ossicle destruction was evident on CT (Figure 1).

**Figure 2:**
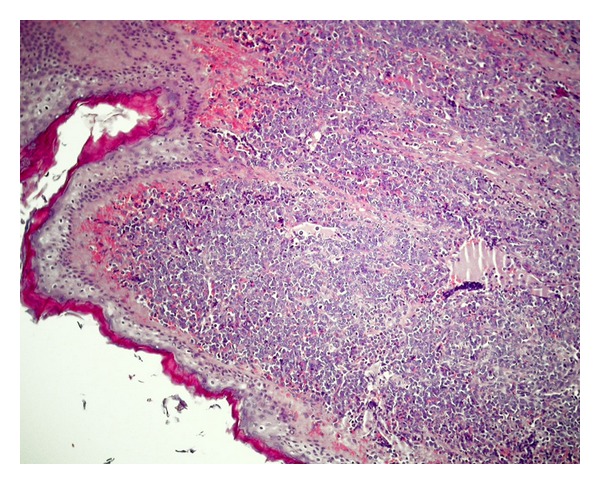
Image of the external auditory canal with epithelial lining and massive cell infiltration of the skin. Haematoxilin-eosine.

**Figure 3:**
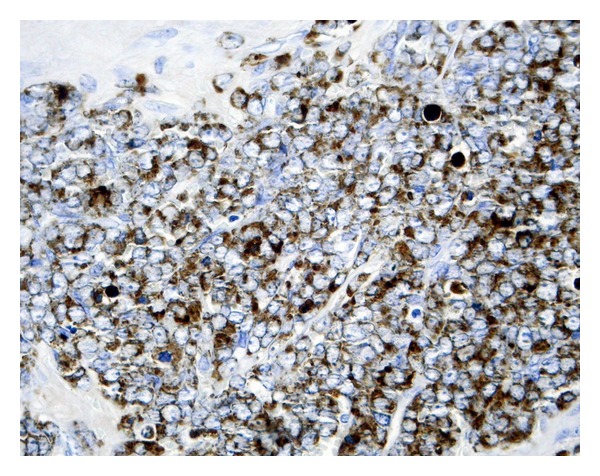
Abundant cytoplasmatic granules, positive for myeloperoxidase. Myeloperoxidase technique.

Under the clinical impression that this patient might have been suffering from atypical mastoiditis and, after having obtained informed consent from him, mastoidectomy and antrectomy were performed opening the posterior attic and preserving untouched the ossicular chain. A soft and friable tissue infiltrated the subcutaneous tissue and filled the mastoid and posterior attic. Peroperative biopsy showed leukaemia infiltration of this tissue. Therefore, more aggressive surgery was not attempted. Final histopathological report showed the tumour to be a granulocytic sarcoma (Figures 2 and 3).

Bone marrow biopsy revealed recurrence of this patient's acute myeloblastic leukaemia, which had identical cytological characteristics than the initial sample.

The patient followed the same chemotherapy regime as in the first occasion, and once more, complete clinical remission was obtained. His otodynia and facial paralysis disappeared and his hearing returned to normal (pure tone audiometry and tympanometry were both normal). His external auditory canal swelling subsided and the appearance of his eardrum was normal. CT scan of the mastoids showed complete disappearance of the abnormal tissue that once had filled the right mastoid.

Later on, this patient underwent autologous-transplantation with haematopoietic stem cells transplantation. On October 2007, his Acute Myeloblastic Leukaemia relapsed and bone marrow transplantation from a non-related donor was performed. Three years later, the patient remains in complete remission.

## 3. Discussion

The incidence of GS in patients suffering from leukaemia is about 3–8% but if the patient suffers from M2 subtype acute myeloid leukaemia, the incidence of GS rises significantly. This variety of acute myeloid leukaemia shows 8;21 [*t*(8;21)] translocation in up to 18% of the cases. This cytogenic abnormality is also the most common one in GS [3]. Approximately 10% of M2 type acute myeloid leukaemia patients will develop GS.

GS affects more frequently children [1]. The incidence is significantly higher in black children of African origin, and particularly so, if the aforementioned translocation is present. 

 GS should be suspected in patients suffering from leukaemia and who present symptoms of acute Otomastoiditis associated with facial paralysis [1, 4, 5]. Imaging diagnostic techniques, such as computerized tomography and magnetic resonance imaging, are not absolutely reliable. Therefore, diagnosis should be established by means of biopsy [1].

Chemotherapy is the best therapeutic option for patients with pseudomastoiditis, facial paralysis, and hearing loss associated to GS. Surgery, other than a biopsy, should be avoided, due to the fact that surgical decompression of the facial nerve and handling of the middle ear ossicular chain offer worse outcome than the appropriate chemotherapy [4–6].
